# Cardiac Angiosarcoma in a 17-Year-Old Female: A Rare Case Report and Literature Review

**DOI:** 10.7759/cureus.89079

**Published:** 2025-07-30

**Authors:** Martin Nguyen, Tai Nguyen, Thao Pham

**Affiliations:** 1 Radiology, West Virginia School of Osteopathic Medicine, Lewisburg, USA; 2 Obstetrics and Gynecology, Hung Vuong Hospital, Ho Chi Minh City, VNM; 3 Radiology, University Medical Center, Ho Chi Minh City, VNM

**Keywords:** cardiac angiosarcoma, cardiac magnetic resonance (cmr), cardiac mass, malignant cardiac tumor, primary cardiac tumors

## Abstract

Primary cardiac angiosarcoma is an extremely rare tumor, especially in pediatric populations (0-18 years), which often carries a poor prognosis due to vague symptoms and its aggressive nature. We present a rare case involving a 17-year-old female, illustrating the diagnostic and therapeutic difficulties posed by this condition in an atypical age group. A 17-year-old female presented with a one-month history of dry cough, exacerbated when lying down, and orthopnea requiring two pillows for sleep. After an unremarkable outpatient evaluation, an echocardiogram revealed a large pericardial effusion with preserved ejection fraction (60%), leading to hospital admission. Computed tomography (CT) identified a 3.0 × 4.0 cm irregular mass invading the right atrium, multiple mediastinal and pulmonary nodules, a solitary 3 cm liver lesion, and a skull mass. Cardiac magnetic resonance imaging (MRI) confirmed a 5.0 × 3.0 × 4.0 cm mass located in the right atrium with hyperintensity on first-pass perfusion imaging, heterogeneous contrast enhancement, and significant pericardial effusion. Liver biopsy confirmed metastatic angiosarcoma, likely of cardiac origin, with positive immunohistochemical markers (CD31, CD34, CD117; Ki-67 40%). The patient underwent six months of chemotherapy (paclitaxel and gemcitabine) without improvement. Follow-up MRI showed disease progression with new liver and kidney lesions. The patient discontinued treatment and died shortly thereafter. Cardiac AS is an aggressive malignancy with nonspecific symptoms, such as dyspnea and cough, complicating early diagnosis. These tumors most often present with distant metastases. Imaging modalities, including echocardiography, CT, and MRI, are critical for diagnosis, with histopathology and immunohistochemistry confirming the diagnosis. Treatment typically involves surgical resection and chemotherapy. However, there is no standard treatment for cardiac angiosarcoma. This case underscores the need for heightened clinical suspicion in young patients with persistent cardiopulmonary symptoms and the urgent need for novel therapeutic strategies to improve outcomes.

## Introduction

Primary cardiac tumors (PCTs) are rare clinical entities with a prevalence of 0.001-0.03%, as reported in autopsy studies [1,2]. Approximately 25% of these tumors are malignant, with undifferentiated sarcomas being the most common, followed by angiosarcomas (AS), leiomyosarcomas, and rhabdomyosarcomas [1]. PCTs are significantly less frequent than cardiac metastases, which are often associated with lung carcinoma [3].

AS is a malignant tumor that originates from vascular or lymphatic endothelial cells and occurs in 0.0017-0.33% of autopsy cases [2]. It often occurs from the third to the fifth decade of life with a male-to-female ratio of about 2:1 to 3:1 [4,5]. At the time of diagnosis, metastasis has occurred in most cases [6].

While cardiac AS is already exceedingly rare overall, its occurrence in adolescents is profoundly unusual, with fewer than 20 pediatric cases (ages 0-18) reported globally in the medical literature over decades [7]. Population-based surveillance from the Surveillance, Epidemiology, and End Results (SEER) database (1973-2011) further underscores this rarity, identifying only 27 malignant PCTs in individuals ≤18 years across 39 years, of which sarcomas represented 19 cases (with AS being the most common histopathological type among sarcomas overall) [8]. This rarity contributes to diagnostic delays, as nonspecific symptoms like cough and orthopnea in teenagers are more commonly attributed to benign causes such as respiratory infections or allergies, rather than prompting suspicion for cardiac tumors. In this case report, we present the case of a 17-year-old female with cardiac AS with metastasis to the lungs and liver, illustrating these diagnostic challenges and their clinical implications.

This case was presented as a poster at the SHM Converge Scientific Abstract Competition, Society of Hospital Medicine (April 22-25, 2025, Las Vegas, NV).

## Case presentation

**Figure 1:**
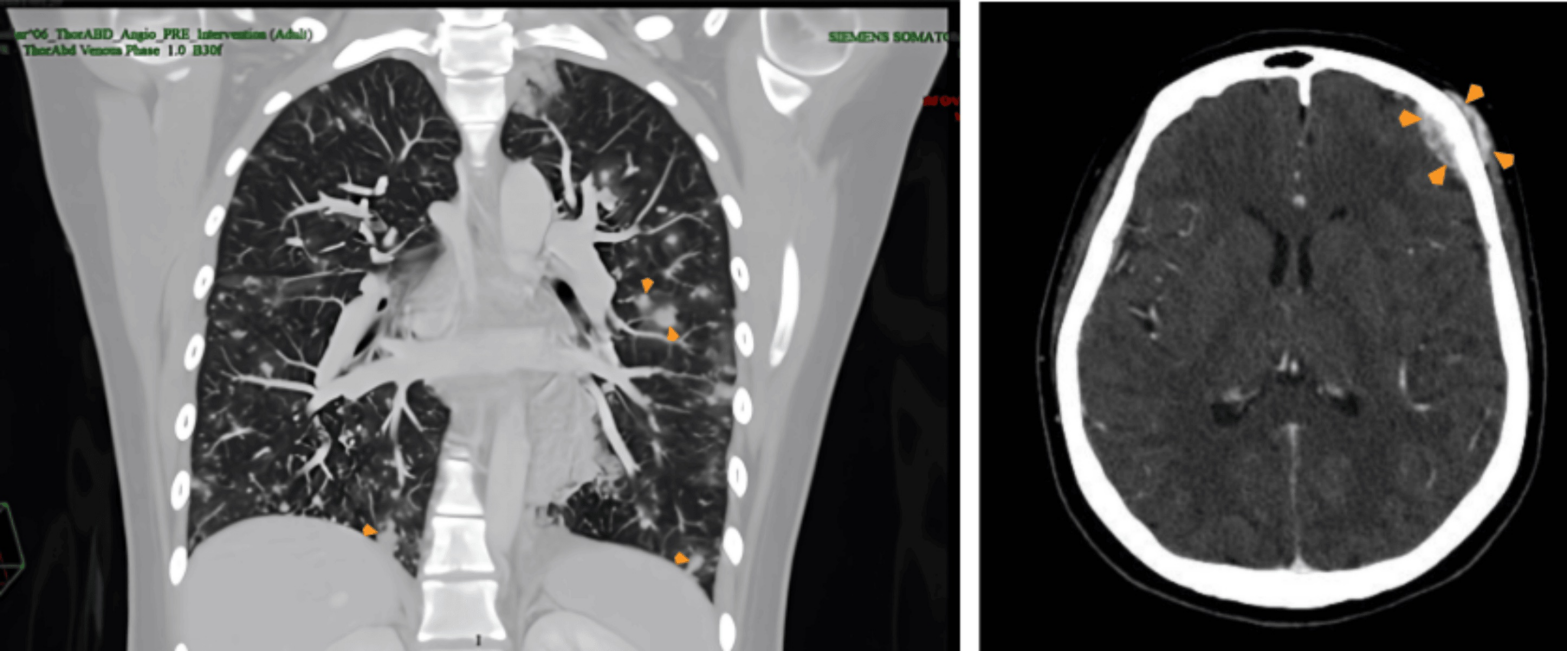
(Left panel) CT scan of lungs and brain on the initial visit with contrast demonstrated multiple nodules in both lungs of different sizes (orange arrowheads). (Right panel) Brain CT scan showing a mass lesion of the skull (orange arrowheads). CT, computed tomography

A 17-year-old female (height, 154 cm; weight, 54 kg; body mass index (BMI), 23.4) presented to the outpatient clinic with a one-month history of dry cough that worsened when lying down and improved upon sitting or standing. She reported needing two pillows to sleep due to discomfort. Two weeks prior, she had visited an ear-nose-throat (ENT) clinic, was diagnosed with pharyngitis, and was treated with antibiotics. However, symptoms persisted. Complete blood count demonstrated mild anemia (Table 1). Other laboratory findings were unremarkable. An echocardiogram was performed and revealed a large pericardial effusion with a preserved ejection fraction (EF) of 60%, prompting immediate hospital admission. Subsequent computed tomography (CT) imaging showed a 3.0 × 4.0 cm mass with irregular borders located in the right atrium (RA), hyperenhancement features, and thickened pericardial membranes with effusion (up to 2.5 cm). Multiple mediastinal nodules were also observed. A solitary 3-cm heterogeneous hyperenhancing liver lesion with ill-defined borders was detected, along with multiple pulmonary nodules and a skull mass (Figure 1).

**Table 1:** Complete blood count on initial visit. ALT, alanine aminotransferase; AST, aspartate aminotransferase; Hb, hemoglobin; Hct, hematocrit; WBC, white blood cell; MCV, mean corpuscular volume; PLT, platelet

	Results	References
WBC	10.01	4-11 (× 10^9^/L)
Hb	11.2	12.1-15.1 g/dL
Hct	34.8	36.1%-44.3%
PLT	493	150-400 (× 10^9^/L)
MCV	87.4	78-100 fL
AST	55	8-33 U/L
ALT	37	4-36 U/L

**Figure 2:**
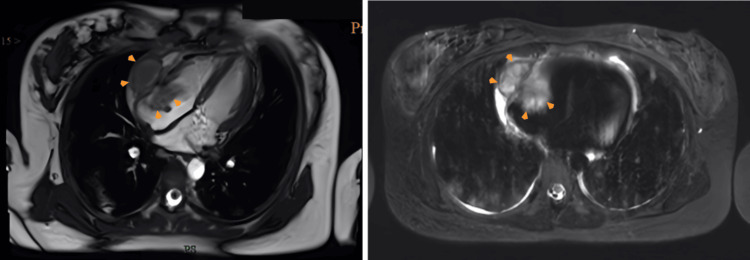
Cardiac MRI. (Left panel) Four-chamber cine imaging. Hyperintensity was demonstrated in the mass region with FS T2-weighted, combined with pericardial effusion. (Right panel) A mass (5 × 3 × 4 cm) (orange arrowheads) is located in the lateral wall of the right atrium (RA) and pericardial space. A portion protruded into the RA cavity. Mass borders are irregular. FS, fat saturation; MRI, magnetic resonance imaging

**Figure 3:**
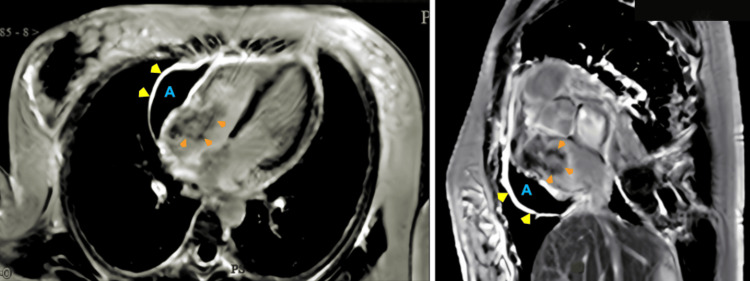
Cardiac MRI, axial and sagittal planes. The pericardial membrane (yellow arrowheads) was thick and enhanced with notable pericardial effusion (letter A). (Left panel) After gadolinium administration, the mass (orange arrowheads) demonstrated hyperenhancement features. (Right panel) Sagittal plane, a significant portion of the mass (orange arrowheads) is located in the RA, with many features suggesting an underlying malignant pathology, including thick pericardium (yellow arrowheads) and pericardial effusion (letter A). MRI, magnetic resonance imaging; RA, right atrium

**Figure 4:**
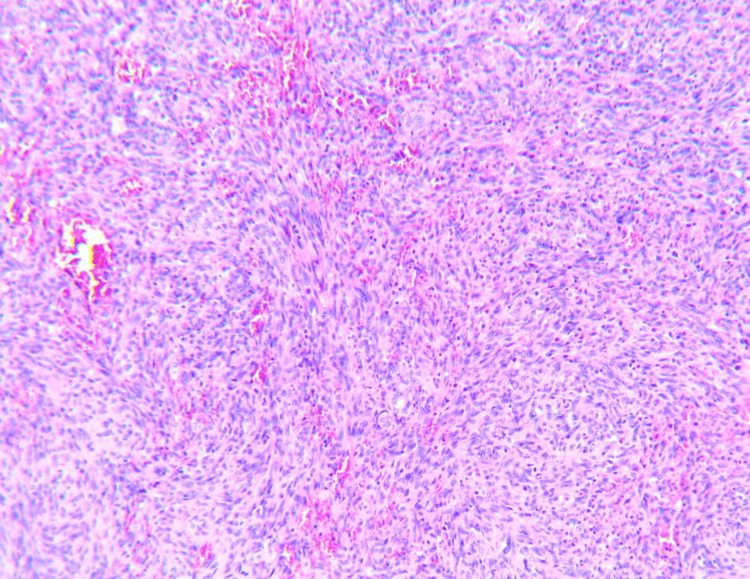
Biopsy of the liver lesions demonstrated the invasion of tumor cells and irregular anastomosing vessels. Stain: hematoxylin and eosin, original magnification ×40

**Figure 5:**
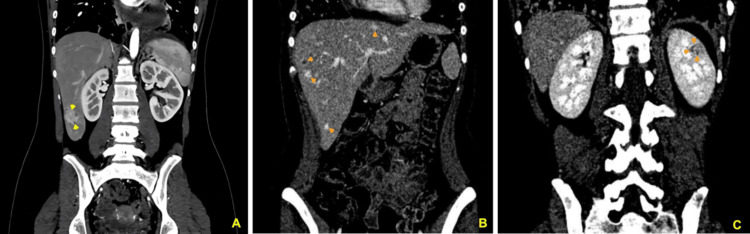
Abdominal CT. (Panel A) A solitary lesion (3.0 × 3.0 cm) (yellow arrowheads) was found in the liver on the initial visit. It had irregular border, hyperenhancement features after contrast administration. (Panels B and C) Follow-up CT after six months of chemotherapy demonstrated multiple nodular lesions (examples include orange arrowheads in Panel B) in the liver and on mass in the left kidney (Panel C, orange arrowheads). CT, computed tomography

Cardiac magnetic resonance imaging (MRI) confirmed a 5.0 × 3.0 × 4.0 cm irregular mass involving the RA and pericardium, with most of the mass occupying the RA lumen (Figures 2-3). The lesion exhibited T2-weighted hyperintensity and heterogeneous T1-weighted signal with internal foci indicative of hemorrhage. It displayed malignant characteristics, including irregular margins, hyperintensity on first-pass perfusion imaging, and heterogeneous contrast enhancement. Additionally, the lesion invaded the tricuspid valve and pericardium, evidenced by pericardial effusion, thickening, and enhancement. There was no evidence of inflow obstruction from RA to the right ventricle. Abdominal CT further identified a 3.0 × 3.0 cm mass in liver segment VI. Despite imaging characteristics strongly suggestive of a PCT, considerations of interventional feasibility, potential hemorrhagic risks, and patient safety prompted a laparoscopic biopsy of the hepatic lesion (Figure 4). Pathological examination confirmed AS, likely metastatic from a cardiac origin. Immunohistochemical markers were obtained for more detailed investigation of the tumor (Table 2). Surgical intervention was deemed inappropriate due to the presence of distant metastases. The patient underwent six months of chemotherapy with paclitaxel and gemcitabine. She underwent an initial cycle of paclitaxel chemotherapy at a dose of 100 mg administered via intravenous (IV) drip once daily for 15 days, followed by a two-week rest period. Subsequently, a second cycle of paclitaxel was initiated at the same dosage and regimen for another 15 days. Following this, the patient was admitted for a planned gemcitabine regimen; however, treatment was postponed due to epistaxis and abnormal vaginal bleeding. Ten days later, the first cycle of gemcitabine was started at a dose of 1000 mg in 100 mL via IV drip once daily for 15 days. One week after completing this cycle, the patient required multiple hospital admissions due to severe fatigue and dyspnea, preventing continuation of further chemotherapy. During the final hospitalization, she developed respiratory failure, severe pneumonia, profound anemia, and evidence of extensive metastasis to multiple organs and bones. Follow-up CT revealed disease progression, with new multiple nodular lesions in the liver and left kidney, indicating systemic metastases (Figure 5). The patient expressed a wish to pass away at home and was discharged upon request, succumbing shortly thereafter, approximately seven months after diagnosis.

**Table 2:** Immunohistochemical results. CD, cluster of differentiation; CK, cytokeratin; C-myc, cellular myelocytomatosis oncogene; DOG1, discovered on gastrointestinal stromal tumors 1; HMB45, human melanoma black-45; SMA, smooth muscle actin

Marker	Result
CD31	Positive
CD34	Positive
CD117	Positive
CK	Negative
DOG1	Negative
SMA	Negative
Desmin	Negative
C-myc	Negative
HMB45	Negative
p53	Negative
Ki-67	40%

## Discussion

The incidence rate of visceral AS is reported to be 300-330 cases per year in the US [9]. The most common locations of visceral AS are the liver (27.6%), heart (13.4%), and bone (12.0%) [9]. Cardiac AS represents the most prevalent malignant primary cardiac neoplasm, characterized by nonspecific symptoms that complicate early diagnosis [6,10]. Its aggressive behavior contributes to a poor prognosis, with metastases present in 47-89% of cases at diagnosis, commonly affecting the lungs, brain, bone, and colon [10,11]. In a case series including six patients of primary cardiac AS, Herrmann et al. [12] reported that the mean survival was six months (ranging from three to 11 months). Symptoms typically occur during the later stages of the disease when metastasis has initiated [10,11]. A review by Strohl et al. [13] of 52 cardiac AS cases found that 86% presented with right-sided congestive heart failure, primarily due to hemorrhagic pericardial tamponade or superior vena cava obstruction, often without prior cardiac history and with abrupt symptom onset. This underscores the challenge of timely diagnosis given the lack of early, specific clinical indicators.

With over 80% of cardiac AS arising in the RA, there are no specific symptoms associated with this pathology [14]. Symptoms and clinical findings depend on tumor location, local invasion, and systemic metastasis, which may include arrhythmia, hemoptysis, fever, coughing, dyspnea, pericardial effusion, and abnormal functioning of cardiac valves [6,15]. Dyspnea is the most frequent symptom, reported in 59-88% of the cases [16]. Electrocardiographic abnormalities, such as arrhythmias, heart block, or nonspecific ST-T wave changes, may also occur [17]. The nonspecific nature of these findings, combined with their dependence on tumor characteristics, emphasizes the need for advanced diagnostic tools to differentiate cardiac AS from other cardiac pathologies and guide clinical management effectively.

Imaging diagnostic modalities for cardiac AS include echocardiogram, CT, and MRI [10,11]. Transthoracic echocardiogram (TTE) is often the initial imaging study due to its wide availability and lower cost [11]. It is helpful in the evaluation of tumor size, shape, mobility, and potential additional lesions in all the cardiac chambers [18]. Transesophageal echocardiography (TEE) can accurately assess the baseline valvular stenosis or regurgitation and guide cannulation preoperatively [18]. The diagnostic sensitivity of TTE and TEE for PCTs is 93% and 97%, respectively [19]. Echocardiographic features of AS typically include an inhomogeneous mass with poorly defined borders, and malignancy is suggested by right-sided lesions, extracardiac invasion, or pericardial effusion [11,20]. In a series of 149 cases of PCTs, Meng et al. [19] reported that the proportion of pericardial involvement in malignant tumors was significantly higher in the malignant tumors versus myxoma tumors (25.8% vs. 0%, p = 0.00) and versus non-myxomatous benign tumors (25.8% vs. 4.7%, p = 0.01). One notable limitation of echocardiography is its dependence on the operator’s experience as well as its inability to characterize different tissue types [11,21].

CT and MRI are better than echocardiography to detect extracardiac involvement as well as metastasis [11]. CT is helpful in calcification detection as well as assisting with transthoracic biopsies [11]. Cardiac MRI is very helpful in the mass characterization and its vascularization [21]. It is essential in the evaluation of abnormalities of the myocardium [11,20,22]. It can differentiate a thrombus from a tumor with the utilization of delayed enhancement of gadolinium [23]. Tumors with their increased vascularity would enhance brightly with this contrast, while thrombi tend to stay unaffected [23]. PCTs often demonstrate heterogeneity on T1-weighted images and hyperintensity on T2-weighted images [11]. Cardiovascular magnetic resonance (CMR) imaging is pivotal for distinguishing benign from malignant cardiac tumors. Malignant tumors are characterized by distinct imaging features, such as right-sided location, sessile and polylobulated morphology, infiltration of adjacent structures, pericardial effusion, first-pass perfusion, and heterogeneous contrast enhancement [24,25]. AS, for instance, typically present with a multilobulated, cauliflower-like appearance and heterogeneous signal intensity on T1- and T2-weighted sequences due to intratumoral necrosis and hemorrhage. These tumors exhibit pronounced, heterogeneous contrast uptake and often invade the pericardium, producing the distinctive “sunray” or “sunburst” enhancement pattern on post-contrast imaging [26,27].

The gold standard in CA diagnosis remains to be histopathology and immunohistochemistry [6]. Immunohistochemical staining shows positive staining for CD31, factor VIII, von Willebrand factor (vWF) [28]. Other markers, such as CD34, cytokeratin (CK), and vimentin, can also be helpful [6]. Cardiac AS also poses a challenge for histological diagnosis. In a review of Rettmar et al. [29], they report that only 38% of cases were correctly diagnosed when patients were alive. Moreover, pericardial fluid was positive for malignant cells in only 3.1% of the cases [29]. Endomyocardial biopsy was performed in four cases, and two of them were falsely negative [29]. The authors suggested that pericardial, pleural, and endomyocardial biopsy, as well as pericardial fluid analysis, frequently yielded false-negative results, which may delay diagnosis and treatment [29]. It was recommended that an open biopsy of primary tumor or secondary tumor deposits should be considered for an accurate diagnosis of intracardiac malignancy [29]. In morphology, these tumors have irregular borders and often show hemorrhagic properties [10]. These tumors usually invade nearby structures such as venae cavae and TV [10]. Higher necrosis and mitotic rates are associated with worse outcomes [17]. On a microscopic level, cardiac AS is characterized by vascular channels formed by malignant cells, and other areas of spindle cells and anaplastic cells [30]. The malignant nature is displayed in the anastomosing vascular channels, spindle cell areas, and foci of endothelial tufting [31].

No standardized treatment exists for cardiac AS, with management typically involving surgical resection when feasible, combined with chemotherapy and, occasionally, radiation therapy [11,21]. Although surgery is the first-line treatment of cardiac AS, complete resection is rarely achievable due to the aggressive nature and rapid progression of this tumor [11,32,33]. Most patients present with technically unresectable diseases and metastasis at diagnosis, resulting in poor outcomes [12,33]. Our patient was not indicated for surgical intervention due to the presence of distant metastasis to the lungs and liver. After resection, local recurrence frequently occurs and accounts for one-third of subsequent mortality in these cases [34].

For metastatic disease, chemotherapy with anthracyclines, taxanes, or ifosfamide may be considered [6]. Our patient was indicated to be treated with paclitaxel and gemcitabine. Paclitaxel has demonstrated some success in the management of adult AS [35]. Gemcitabine was added afterward as it can be used as a second-line treatment in metastatic soft tissue sarcomas [36]. In a metastatic case of cardiac AS, Koo et al. [7] reported to treat the patient with multimodal therapy in a 10-year-old patient. He had a RA (6.8 cm) mass obstructing the TV inflow, combined with a large pericardial effusion and presumed pulmonary metastases [7]. He underwent emergent surgical debulking, and biopsy demonstrated cardiac AS. He was initiated on chemotherapy, including paclitaxel and bevacizumab (four cycles). Then he was treated with doxorubicin and ifosfamide for five cycles (anthracycline cumulative dose 375 mg/m^2^). He was then switched to gemcitabine and docetaxel, and then to gemcitabine and nanoparticle albumin-bound (nab) paclitaxel. He was finally indicated pazopanib due to the toxicities of gemcitabine and nab-paclitaxel. However, pazopanib was also stopped due to significant adverse effects. He experienced multiple dose reductions and interruptions of pazopanib because of myelosuppression, acute kidney injury (AKI), and proteinuria. Surgical resection, systemic chemotherapy, and radiation, combined with targeted therapies (pazopanib and bevacizumab), were applied. He was alive five years after diagnosis, making this one of the rare cases in the published pediatric literature with significant success with multimodal therapy for cardiac AS, according to the authors [7].

In a review of 24 patients with cardiac AS treated with surgical excision, the median survival time was 10 months, regardless of adjuvant therapy [12]. However, some case reports described better outcomes. Sørlie et al. [37] reported a case treated with surgical resection followed by radiotherapy, with no signs of recurrence or metastasis 36 months post-surgery. Fukunaga et al. [38] reported a case of cardiac AS in an 18-year-old girl managed by surgical excision followed by radiochemotherapy. She died from AS metastases more than three years after surgery. Although AS originates from vascular endothelium, anti-vascular endothelial growth factor (anti-VEGF) therapy with bevacizumab was not proven to have benefits [7,39]. Leading causes of mortality include myocardial invasion, cardiac tamponade, metastasis, arrhythmias, and embolism [40].

To investigate the prognosis of patients with metastatic cardiac AS, we conducted a literature review on the PubMed database to search for similar cases of metastatic cardiac AS published in the pediatric population (Table 3). We used these terms and their combinations, including “cardiac angiosarcoma”, “primary cardiac angiosarcoma”, “metastatic cardiac angiosarcoma”, and “case report”. Inclusion criteria were defined as cases with both primary and metastatic tumor sites. Cases without biopsy results or those outside the pediatric age range (0-18 years) were excluded. Cardiac AS originated from the RA in most cases and was most likely to metastasize to the lungs. In general, the prognosis is poor, with many patients dying shortly after diagnosis.

**Table 3:** Pediatric cardiac angiosarcoma cases (ages 0-18). This table compiles all identifiable, unique, biopsy-confirmed cases of primary cardiac angiosarcoma in pediatric patients from PubMed literature (1970-2024). It includes key clinical details derived from abstracts and full texts (where applicable). Total: 14 unique cases, reflecting the tumor's rarity. Chemo, chemotherapy; Dx, diagnosis; LN, lymph node; RA, right atrium; rad, radiation therapy; Tx, treatment; NS, not specified (e.g., due to limited details in abstracts or full-texts); RT, radiotherapy. Cases are sorted chronologically by publication year.

Authors	Year	Patient age, sex	Primary site	Metastasis	Outcome	Tx modality
Wakely et al. [45]	1987	15 years, male	RA	NS	NS	NS
Nakamichi et al. [46]	1997	Eight years, female	RA	None	Alive 53 months post-op	Surgical excision + two years multidisciplinary therapy (no chemo details)
Booth et al. [43]	2001	23 months, female	Heart	Brain, ovaries, bone marrow	Died eight months post-presentation	Surgical biopsy (no chemo details)
Lundkvist et al. [44]	2002	Pediatric NS (siblings)	Heart	NS	Died shortly after Dx	NS
du Toit-Prinsloo et al. [47]	2016	16 years, male	RA	None	Sudden death	None (postmortem diagnosis)
Fukunaga et al. [38]	2017	18 years, female	RA	None initially; then metastasized to liver	Died three years post-Dx	Surgical excision + radiochemotherapy (no specific dosages provided).
Citak et al. [48]	2018	15 years, female	RA	Mediastinal LN, left kidney	Recurrence at eight months	Surgery (partial resection + pericardial patch); chemo (ifosfamide + doxorubicin, six cycles); RT (54 Gy); post-recurrence chemo (gemcitabine + docetaxel)
Hod et al. [49]	2018	18 years, female	Heart	Pulmonary	NS	NS
Koo et al. [7]	2021	10 years, male	Heart	Metastatic NS	Alive five years post-Dx (longest survivor)	Surgical resection; conventional chemo; rad; targeted (bevacizumab, pazopanib; no dosages)
Maliszewska et al. [50]	2023	12 years, male	Heart	Lungs, mediastinal LN	NS	Docetaxel (30 mg/m², 45 mg total, one-hour IV infusion)
Farzin et al. [51]	2023	17 years, female	Pericardium	NS	Died of respiratory failure	Total pericardiectomy + chemo (two weeks; no dosages)
Lahmouch et al. [52]	2024	Teenage, female	RA	NS	NS	NS
Sharrack et al. [53]	2024	17 years, male	Heart	NS	Tumor growth post-biopsy	Percutaneous + surgical biopsy (no chemo)
Nguyen et al. (current case)	2025	17 years, female	RA	Lungs, liver, skull, and kidney	Died seven months post-Dx	Chemo (paclitaxel + gemcitabine, six months). Dose: paclitaxel 100 mg once daily (two cycles), then gemcitabine 1000 mg (IV drip) once daily (two cycles).

Heart transplantation has been performed as a potential treatment for cardiac AS. A study by Li et al. [41] of six heart transplant cases in a Chinese hospital found no significant survival difference between transplant and palliative groups (p = 0.768), suggesting that cardiac AS may not be a suitable indication for transplantation. Adjuvant chemotherapy after transplantation was not associated with survival benefits [41]. Overall, heart transplantation has not demonstrated favorable outcomes for this malignancy [42]. The aggressive nature of cardiac AS, coupled with diagnostic and therapeutic challenges, underscores the urgent need for research into more effective diagnostic tools and targeted therapies to improve patient outcomes.

## Conclusions

Cardiac AS is a rare and aggressive malignancy with nonspecific symptoms that frequently result in delayed diagnosis and metastatic presentation at the time of detection, posing significant management challenges. Clinicians should maintain a high index of suspicion, particularly in younger patients exhibiting persistent dyspnea, chronic cough, or recurrent pericardial effusions of unknown origin. Early imaging with echocardiography, CT, and MRI is vital for timely detection and intervention, though diagnostic challenges persist despite technological advancements, underscoring the need for prompt recognition to enhance outcomes.

Among cardiac sarcomas, AS confers the poorest prognosis. Surgical resection remains the cornerstone of treatment when feasible, supplemented by neoadjuvant chemotherapy to reduce tumor burden preoperatively, alongside radiotherapy or targeted therapies in select cases. Given the disease's rarity, larger multicenter studies and randomized trials are essential to evaluate emerging modalities, including immunotherapy and genetically tailored approaches, for improved survival.
